# Value chain approaches to reducing policy spillovers on international business

**DOI:** 10.1057/s42214-020-00083-5

**Published:** 2020-11-27

**Authors:** Christopher Findlay, Bernard Hoekman

**Affiliations:** 1 Crawford School, Australian National University, Canberra, Australia; 2 Robert Schuman Centre for Advanced Studies, European University Institute and CEPR, Florence, Italy

**Keywords:** global value chains, global production networks, international business, trade regulation, multi-stakeholder initiatives, public–private policy partnerships

## Abstract

Government policy can add to the costs of doing international business. It can distort the construction of and raise the costs of operation of global value chains (GVCs), to the detriment of the participating economies. Given rising technological and market-driven headwinds confronting GVCs, countries seeking to attract GVC activities have greater incentives to identify and address policies that negatively affect international business investment. Cooperation of businesses with regulators, analysts, and researchers has the scope to develop better policy. This paper suggests principles for the design and operation of such cooperation, drawing on the experience with multi-stakeholder value chain partnerships and the policy responses to the 2020 COVID-19 pandemic.

## INTRODUCTION

Global value chains (GVCs) offer significant benefits to participants and final consumers, allowing firms or plants in different geographic locations to specialize in tasks that are needed to produce a good or service. GVCs account for some 50% of global trade (World Bank, 2020). A variety of domestic policies in the countries where GVC activities occur may support or impede their operation. Insofar as policies are perceived as constraints by value chain managers, they will influence the design and intensity of the use of GVCs, in the process diminishing the benefits available (Wang, Gilland, & Tomlin, 2011). Efforts may be made by firms to engage with the relevant authorities regarding policies perceived to act as impediments, or, alternatively, to identify areas where policies are needed, but given the range of measures which might apply, the outcome is often piecemeal and runs the risk of being ineffective.

Identifying the sources and incidence of policy frictions that add to the costs of international trade is complex. One response to this issue, proposed in the trade policy literature, is to construct ‘supply chain councils’ (Hoekman, 2013; World Economic Forum, 2013) that bring together the key actors involved in, and concerned with, the operation of GVCs to identify frictions and determine whether they are due to – or can be reduced by changes in – policy measures. The concept has parallels with public–private policy dialogue platforms, online knowledge portals and national or regional roundtables that have been established by international organizations to pursue environmental sustainability, and social and corporate social responsibility objectives.^1^ It is also related to multi-stakeholder initiatives focused on private governance of value chains that promote dialogue between private actors involved in, or affected by, specific chains. The latter may seek to establish agreement on voluntary production standards, certification of producers, and monitoring of implementation. Such ‘value chain partnerships’ (Bitzer & Glasbergen, 2015) have mostly focused on forestry and agricultural products (such as soy and palm oil) but have also been applied to specific textile and apparel and electronics value chains.^2^

The concept of value chain platforms used in this paper differs from such mechanisms in focusing on the effects of domestic and foreign policies, in particular their impact on the design and operation of value chains.^3^ The purpose is to facilitate and frame deliberation and cooperation among the relevant government agencies that regulate GVCs, the actors that operate and are affected by them, and the research community to identify options to reduce the incidence of value chain frictions. What the value chain approach adds to extant national business–government dialogue mechanisms is its multi-sector, cross-cutting nature, spanning all the significant activities associated with a global production network and leveraging the knowledge of the firms involved as suppliers and buyers, and the expertise of analysts coming from different academic disciplines and practice.^4^

The contribution of this paper is to lay out the case for public–private mechanisms for policy dialogue centered around GVCs. Such mechanisms complement both the informational lobbying and advocacy activities of companies and their engagement in sustainability and corporate social responsibility governance initiatives, which have become a major feature of international business–government–NGO relations (Boddewyn, 2016). We conceive of value chain platforms as instruments that operate at both the national and international level to improve the efficiency and reliability (robustness) of international production networks and to help governments attain non-economic and regulatory objectives. The salience of the concept and its potential utility is illustrated by policy responses to the COVID-19 pandemic, with many countries imposing export controls for medical and food products to maximize domestic supply, in the process impeding supply responses. Mechanisms establishing a solid basis for sharing information on the organization and operation of global value chains for protective equipment and medical supplies would have helped create a common understanding of how to best ramp up supply.

The plan of the paper is as follows. In “Global Value Chains” we first briefly review the nature of GVCs. “Regulatory Heterogeneity, International Trade Costs, and GVCs” discusses how policies affect the operations of GVCs. “Identifying Policy Dimensions of Value Chain Frictions” and “Public–Private Value Chain Platforms” discuss the concept of taking a GVC approach to policy design and implementation, outlining how value chain platforms might operate and their benefits to business, and from business, in its engagement. “Examples of Operationalizing the Concept” discusses several examples of transnational value chain partnerships and public–private trade dialogue mechanisms. “Learning from COVID-19 Policy Responses” discusses the salience of the concept in assisting governments and international business respond to global demand and/or supply shocks, reflecting on the trade policy responses of governments to the COVID-19 pandemic. “Conclusion” concludes.

## GLOBAL VALUE CHAINS

The data suggest that, since the 2008–09 financial crisis, there has been a decline in the intensity of GVCs. Miroudot and Nordstrom (2020) use the 2018 update of the OECD Trade in Value-Added (TiVA) database to measure the number of domestic and foreign production stages in GVCs, the frequency of border crossings and the geographic length. They find that the cross-border intensity of GVCs peaked in 2012, after which the chains started becoming more domestic or regional. They estimate that the reduction in the average length of chains since 2012 is 50 km per year.

This shortening of GVCs may reflect a mix of factors, including a less hospitable policy environment, reflected in increasing protectionism and associated policy uncertainty (Constantinescu, Mattoo, and Ruta 2020a, b), and technological, market, and managerial forces that affect the incentives to offshore and to re-shore production activities (Barbieri, Ciabuschi, Fratocchi, & Vignoli, 2018; De Backer et al., 2016). Of particular salience to this paper is the increase in national trade policy activism since the late 2000s.^5^ Evenett (2019) makes a ‘call to arms’ in this journal to scholars of international business to make greater use of the better data available on government responses complied since 2008, observing that the measures applied tend not to be traditional trade measures but ‘murkier (less transparent) forms of state discrimination’ (p. 29). These can sit within the processes of designing and implementing standards and other forms of regulation. International business gains from a response to this trend, and the proposal made here offers a channel to do so. Evenett (2020) observes that the forms of policy response may vary by crisis, and that a feature of recent events has been the use of restrictions on exports (e.g., of or related to medical products). Restrictions of these types impede successful operations of GVCs.

Value chains have long been recognized in the international business literature. Porter (1985) argued that an analysis of the origins of a firm’s value was important to understand its competitive advantage. The chain which generated value consisted of a set of primary activities, such as procuring inputs, converting them to another form and delivering them to customers, and support activities, such as the firm’s infrastructure, research and development, and people management systems. The overall structure was referred to as a value chain since the goal was to produce greater value for the final buyers in a product or service. The concept was taken further by Gary Gereffi, who has made a significant contribution over time to the analysis of the operations of GVCs (see for example Gereffi, 1999; Gereffi, Humphrey and Sturgeon, 2005; Gereffi and Fernandez-Stark, 2011). Gereffi (2019) and Pananond, Gereffi and Pedersen (2020) provide a framework to connect research on global strategy with that on global value chains and discuss the scope to more closely connect research in international business and international economics, a theme we elaborate on in this paper.^6^

The economic implications of GVCs has attracted considerable attention from researchers and international agencies, which have documented that gains from participation in these chains include productivity growth, employment, and rising incomes.^7^ A basic feature of a GVC is that part, and often much, of the value added embodied in a product reflects inputs sourced from different countries. Locations for activities are chosen according to their competitiveness in completing a task, which reduces the cost of the final product or service. GVCs have different shapes, such as a snake (in which a sequence of activities occurs) or a network (where activities take place at the same time to be combined at a central point). The chains are managed by a key player (“lead firm”) whose location in terms of distance to the final consumer varies. Some chains involve many countries, but the same process of consideration of ‘where best to do what’ applies even within a bilateral setting. GVCs require many services inputs, which are elements of Porter’s concept of support activities. GVCs involve flows of foreign direct investment (FDI),^8^ which often embodies technology and know-how, movements of data and of people, and financial flows.

**Figure 1:**
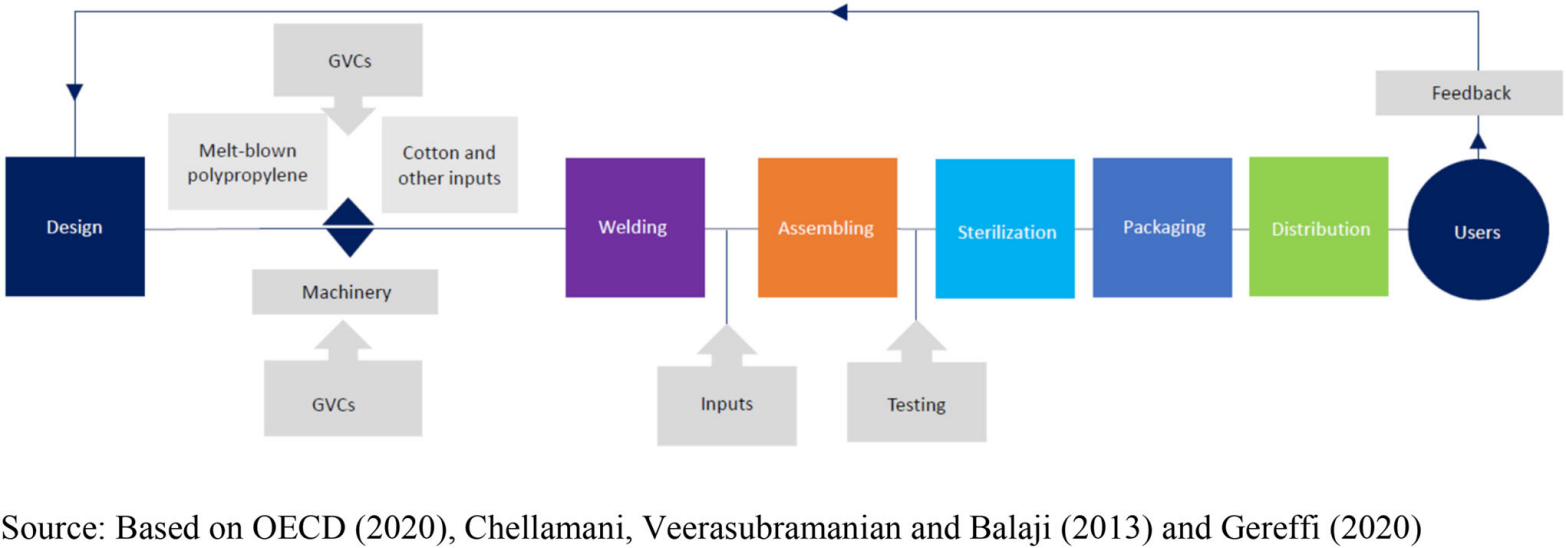
Global value chain for face masks.

We offer an example of a GVC, leading to the production of face masks, the operation of which has attracted attention during the COVID-19 pandemic.^9^ This chain is summarized in Figure 1. The main stages involve design, sourcing of inputs to be processed, procurement of machines for that purpose, then assembly, sterilization, testing, packaging, labelling and distribution. Inputs include non-woven fibers (‘melt-blown polypropylene’), which have their own value chain. Assembly involves ultrasonic welding of three layers – cotton in contact with the mouth, the melt-blown material that captures small particles, and an outer layer to catch liquid splashes. This complex step is followed by relatively simple steps of attachments such as metal strips, elastic, cotton etc., to the welded layers.^10^

There are machines (which have their own GVCs) that integrate welding and assembly but welding and final assembly could also be separated. The chain up to the point of welding (or final assembly) could take the form of a network, which includes processes of testing involving different and specialist machinery. Following assembly is sterilization, packaging and labeling, and distribution. Different categories of masks may be produced targeting different applications. The value chain involves important feedback from users into (re-)design, to reflect customer experience of mask characteristics such as comfort and effectiveness, leading to the creation of further value and a greater willingness to pay.

Not shown in Figure 1 are the data flows involved in coordination of the process or the financial flows that are required. Figure 1 is agnostic about the location of the activities in the chain but, as the dividers between the activities in the figure indicate, they could be located across several countries, including those which differ from the location of the final consumer. It is also agnostic about the location of the lead firms that manage the GVC. In 2017, the top three exporters of face masks in final product form were China (41%), the US (18%), and Germany (7%) (OECD, 2020).

## REGULATORY HETEROGENEITY, INTERNATIONAL TRADE COSTS, AND GVCS

The design of GVCs will be influenced by the ease of moving goods and services within the GVC across borders. As traditional trade barriers that constrain market access such as tariffs have been reduced, policies of a regulatory nature – nontariff measures (NTMs) – have become more prominent as factors affecting the operation of GVCs. Examples are product regulation to achieve health, safety, or security objectives, licensing requirements, certification and conformity assessment procedures, data privacy, and consumer protection rules. The motivation of the application of these measures need not be to protect local firms, but even if the motivation for product or production process regulation is similar across countries, and national policies apply equally to local and foreign firms and products, a multiplicity of regulatory norms and related enforcement requirements in different countries implies increased costs for international business (Hoekman & Sabel, 2019). Even if the substance of regulation is very similar across countries, duplicative testing and certification requirements imply (redundant) additional costs for international firms. Extensive empirical research has shown that the economic impacts of NTMs often exceed border barriers such as tariffs (Francois & Hoekman, 2019).

We illustrate the impact of these sorts of barriers by reference to the GVC in face masks. We take the regulations of the European Union, which apply to personal protective equipment (EU, 2016), as a guide to the types of standards which apply and to which conformity must be established. These include a set of ‘essential’ health and safety measures, including ergonomics, levels of protection, innocuousness, and comfort, plus many additional requirements. These must be demonstrated in the design stage of production, through the presentation of documentation. The manufacturer must show how their intended production process will lead to a product that will meet the design specifications. There is also a role for a conformity assessment procedure in which an approved agency examines both the design specification and manufacturing process as well as testing a specimen, and which then issues a certificate. Materials used in the production process must also be consistent with various standards, related for example to the consequences of exposure to water or fire, or ease of respiration. In the distribution stage, businesses involved are expected to comply with a monitoring process (‘market surveillance’), which checks that all the requirements are complied with. Products must be labeled clearly, and operating instructions are to be supplied.

**Figure 2:**
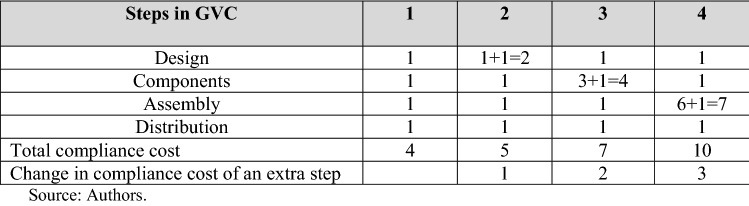
Impact of heterogenous standards in a GVC.

Figure 2 illustrates the impact of regulatory standards and compliance mechanisms in the context of a GVC. Suppose that the GVC involves four stages of design, component sourcing, assembly, and distribution (which may include repackaging and labeling), each of which could take place in a different country. To take an extreme case, we assume that the standards relevant for each stage vary between each country.

In the first column, all production takes place in one country. There are four steps in which compliance must be demonstrated. We assume that the cost involved is fixed and independent of the volume of production. We assign a value of 1 to the fixed costs of compliance. In that case, with no use of the GVC, the compliance cost is 4. Moving to the right, suppose that following the design step the rest of the production process takes place in another economy. Producers in that economy procure the design from its provider and establish its conformity with local standards. However, that cost of procurement already includes the compliance cost of meeting the standards of the originating economy. The total compliance cost at the first stage is then 2 and the total cost of compliance rises to 5. The sequence continues, and at each movement, the cost incurred includes all previous compliance costs plus those of demonstrating conformance to local standards at each stage. In the movement from one to three steps in the GVC, the total compliance cost rises from 4 to 10. The compliance cost rises at each stage according to the number of steps in the GVC, that is, the incremental cost of compliance increases with the number of stages.^11^ The benefit of the design of a GVC is via its contribution to access to lower costs of production in different locations for particular steps in the process, but that advantage is offset by these costs of compliance, which are additional to any tariffs and customs clearance-related costs incurred in moving consignments across international borders. As a result, the construction of the GVC will be impeded.

Figure 2 also illustrates that the costs of compliance could be reduced if the differences in regulatory regimes could be removed, e.g., through harmonization or mutual recognition or equivalence regimes. For example, at the other extreme, suppose all standards for each stage for each country were the same, and that each country accepted the compliance testing process of the other. Then, no matter the number of stages involved in the GVC, the total compliance cost would be 4. If there are three steps, the compliance cost is reduced by 60% (from 10 to 4).^12^

The importance of this type of cost was illustrated in the COVID-19 pandemic. Hoekman, Fiorini and Yildirim (2020) report an example of an unforeseen imposition of standards by China, which in April 2020 imposed export controls on medical supplies and protective equipment that did not meet domestic standards. This created major frictions for firms that produced for export and had not been serving the domestic market. As we discuss further in “Examples of Operationalizing the Concept”, closer cooperation among regulators and the private sector through a value chain platform could have helped avoid this situation.

## IDENTIFYING POLICY DIMENSIONS OF VALUE CHAIN FRICTIONS

The rising salience of differences in regulatory regimes and policies, and the foregoing illustration of their potential significance, as a factor impacting trade and operating costs raises a question for international business: how best to engage with governments and societal groups to improve the cost efficiency of regulatory enforcement processes? What nonmarket strategies and corporate political activities will be most effective in reducing the costs of international policy heterogeneity and related policy uncertainty?

Major components of the nonmarket strategies of international businesses involve informational lobbying and supporting the negotiation and implementation of various forms of trade and investment agreements and international regulatory cooperation, but a challenge for international business is the variety of policy instruments which are relevant to the establishment and shape of value chains and their operations. This follows from the range of forms of activities and tasks involved in the value chain operating across borders – people, data, goods, intellectual property, capital, and especially FDI and services. International trade negotiations, the main instrument used by governments to steer cooperation on policies affecting the flow of goods and services, tend to reflect a “silo approach”. Specific policy areas are dealt with in isolation – such as product standards; customs valuation, import licensing; tariffs; or subsidies. Yet business strategy considers all these elements, just one of which could be a “deal breaker”. A more comprehensive approach spanning all the relevant areas of regulation affecting production and trade can be more effective, compared to a narrower focus. For example, an agreement on data privacy may have little impact if other policies that affect cross-border digital exchange are ignored.

Making progress in this respect faces several constraints. Regulations may be set and enforced by different levels of government. Moreover, regulatory policies frequently pertain to “sensitive” sectors and activities where polities have strongly held non-economic preferences – as is the case in a number of regulatory areas relating to privacy and public health for example, where there are significant differences in risk attitudes across countries. In such cases, making progress in reducing differences in regulation is likely to be difficult. A necessary condition for moving forward in reducing regulatory compliance costs and eliminating duplicative and redundant requirements is mutual trust and understanding of regulatory systems and compliance mechanisms in different countries. These matters require communication and cooperation between regulatory authorities.

A further complication is that the prevalence of regulatory standards-setting by non-governmental actors has also grown substantially. This includes voluntary cross-border regulatory standards-setting initiatives, which also attract business engagement. These initiatives often involve non-state actors, including nongovernmental organizations, as well as multinational companies. An example is the GlobalG.A.P.^13^ norms for agricultural products, which become mandatory if importers and retailers require them as a precondition for sourcing decisions. Nongovernmental actors increasingly seek to fill global governance gaps in areas such as the environment and labor standards, sustainability, safety of products, and corporate governance. In addition, the activities of intergovernmental bodies are relevant, such as those of APEC and the OECD to define and agree on good regulatory practices and international standardization.^14^

These considerations help explain why trade agreements focusing on the removal of discriminatory policies (tariffs, quotas) have yet to play a major role in addressing regulatory sources of GVC frictions (Hoekman & Sabel, 2019). Other forms of international regulatory cooperation have in practice been more prominent or prevalent. These range from international standardization (harmonization) to soft law forms of voluntary cooperation among regulators. A major policy question – one that is important for the nonmarket strategies of international businesses – is when and how trade agreements could become more effective vehicles to reduce the costs of regulatory differences across markets. This is arguably a key question from the perspective of design of corporate strategies that apply in a regime of regulatory heterogeneity. The presumption in what follows is that such strategies and associated corporate political activity need to be guided by better analysis of where trade agreements can and cannot help, and where complementary instruments are needed.

Rather than the traditional emphasis on direct lobbying and formulation of business positions regarding the desirable content of trade and investment agreements, a greater focus on supporting the use of – participation in – deliberative mechanisms that bring together key stakeholders – regulators, government officials, business and users/consumer groups – to assess the impacts of regulatory policy regimes and identify potentially beneficial reforms could help establish a path towards lowering or removing unnecessary trade costs. By including those most concerned with regulations – representatives of consumer groups and regulatory bodies – deliberations would ensure greater balancing of cost-based concerns of business against the objectives that motivate regulatory regimes.

We refer to these mechanisms as multi-stakeholder initiatives. While they are more complex to manage and to motivate for boardroom approval, traditional lobbying activities undertaken by government affairs departments tend to generate suspicion on the part of nongovernmental advocacy organizations and counter-arguments that what business identifies as constraints are in fact legitimate and important regulatory measures that are in the public interest. Engagement with other stakeholders centered around value chains can ameliorate such concerns and is more likely to facilitate the needed “ownership” and political support for reforms that reduce costs without undercutting attainment of underlying regulatory goals.

As illustrated in Figure 2, such deliberations should be based on a “whole of chain” approach towards assessing the effects of regulatory policies for pertinent activities across countries. This implies a focus on both upstream (e.g., raw materials; parts; components) and downstream (e.g., distribution) elements as opposed limiting attention the manufacturing or assembly process in each location. In practice, regulatory policies may have a significant impact on trade and production costs for a broad cross section of firms that are linked together as suppliers or buyers in global value chains and production networks. Whatever the ownership and control relationships, the interests of many firms will be correlated across value chains. This is distinct from the industry or product-specific focus that tends to frame thinking of regulators, governments, and civil society when considering the implications of trade agreements. Framing analysis and arguments around the “network” effects of regulatory heterogeneity – impacting on clusters of firms in very different industries that depend on exporting and importing – may help build greater awareness and support for international regulatory cooperation. Ensuring that the process considers seriously the underlying regulatory objectives and supports their attainment is of critical importance.

The manner of constructing the process that has been presented here is now considered in more detail. This is followed in “Examples of Operationalizing the Concept” with some examples of multi-stakeholder trade cost reduction initiatives.

## PUBLIC–PRIVATE VALUE CHAIN PLATFORMS

The World Economic Forum (2013) and Hoekman (2013) propose a mechanism in which governments work with business and analysts to identify what package of reforms would help “tip” value chain investments and growth. In the first instance, attention would focus on several key value chains for a country. The aim would be to develop details of action plans for policy reform and other measures targeting significant GVC frictions. Performance indicators would be designed to monitor implementation of those plans. Given the nature of global production networks, success would require coordination across countries. Hoekman (2014) therefore proposes the creation of a series of international (value) chain councils. They would have a mandate to provide information, to identify the key policy constraints. They would also identify the costs of these policies, for example, how they led to less efficient shapes of the chains or to costly strategies within the chains (such as higher levels of stocks) to manage the uncertainties in the application of policies.

Assistance of researchers may be required to identify these costs. They have access to methodologies that the business sector and policy makers may not. Researchers also play another role. Some businesses who are exporters may have knowledge of impediments but also have the capacity to avoid them, albeit at some cost. The removal of the impediments could have the consequence of increasing the level of competition in export markets, to the detriment of those who have learned or invested in how to jump the barriers while continuing to earn profits. These are also likely to be larger firms, or those who are more productive and can bear the costs of responding to impediments to trade. For example, it is possible that firms already operating behind those impediments and benefiting from them because of the protection they offer from competitors, may be unwilling to share their intelligence. Kim and Osgood (2019) argue that a firm-level perspective leads to a deeper understanding of why different types of firms take various positions on policies related to economic integration. However, in a global value chain context, where the barriers are cost- rather than rent-creating, firms have a stronger incentive to seek their removal. Researchers can contribute to the construction of this argument and provide assessments of the extent of the burdens involved.

Another reason that businesses may be reluctant to share information about impediments related to government practice is because their identity may also be revealed in the process of providing that information, which they may be concerned would lead to a future penalty for them through the reaction by governments, either home or offshore. To avoid these impacts, research institutes can play a valuable intermediary role, aggregating and anonymizing data used for analysis, ensuring that the focus is on stages and tasks of value chains and assessment of the effects of policies on different activities that are undertaken by or affect actors and stakeholders involved in various stages, without identifying individual firms.

In many cases, the issues constraining the development of value chains is the approach taken by governments to matters such as regulation and standards. There may be differences between economies, not for reasons of deliberately seeking to be different and to protect producers based in the home country, but because of different histories or ways of thinking about how to respond to market failures. Issues related to standards include not just the differences between economies but also the way compliance with standards is tested and reported. Issues related to regulation can be divided into those affecting establishment (e.g., licensing, which might apply to domestic and foreign firms, rules on foreign equity, and on options to establish branches or subsidiaries) and on operations (such hiring local staff, areas of business – products, services, or locations). These sorts of barriers can be resolved by establishing, for example, that processes in trading partner economies are equivalent. This requires a common understanding of the purpose of the regulation, that is, that nature of the problem to be solved, the options for responding to that problem and the best choice in the local conditions (there may not be one answer globally to the question of “what is best practice”). An important observation is that these questions are not the elements of negotiation, since they are not amenable to the exchange at the margin of changes in policy (as, in contrast, occurs in negotiations on tariff rates, for example).

Progress with respect to these issues can be made by completing a series of tasks:

1. Improve the transparency of applied policies, which depends on dialogue and learning.
2. Undertake independent analysis of policies, their purpose and their consequences.
3. Consult and exchange information regarding the objectives of regulatory policies and the regulatory regime that is used in each country to implement national policies.
4. Identify alternatives to current policy and the nature of good practice.
5. Identify the constraints on reform, including those reflecting political economy forces, and options (based on experience) for responding.

Business support and engagement will be a critical input and a key differentiator from extant mechanisms through which governments are lobbied and consult stakeholders. Business participants (buyers and sellers along a chain) offer key insights into the seriousness of the impacts of the various policies which affect chain design and operations. They hold the knowledge and materials on which analysts can add value by extrapolating the costs involved in ameliorating strategies taken by business. The value to business from engagement is greater rapid progress in dealing with issues which are constraints on the application of their business models. There is scope for significant returns to effort and, with the right design, the benefits for smaller firms can be significant. Including user businesses as well as supplier businesses in addition to other relevant stakeholders in sustained and regular engagement over time is important, in part because of the complexity of policy-GVC effects but also because market conditions and technology will continue to change.

There is a “knock-on” effect. Explicit adoption of a value chain approach could be particularly useful in the design and pursuit of trade negotiations by targeting cooperation on matters that would make a greater difference from an operational business perspective, thereby helping to mobilize business support for such efforts. Business interest in WTO negotiations has been waning over the last decade, with more attention and resources shifting to regional integration efforts. While regional integration is a potentially important source of trade cost reduction for firms, they are by definition (construction) much more limited than what could be realized through multilateral agreements. More rapid progress in global trade talks offers a wider set of benefits to business, especially in the context of the current uncertainties in the world trading system.^15^

Successful international agreements addressing regulatory policies such as the WTO agreements on sanitary and phytosanitary measures, technical barriers to trade and trade facilitation are all associated with a body of agreed technical knowledge and accumulated good will among the relevant national regulatory agencies. Haas (1992) refers to a group of stakeholders and experts linked in this way as an epistemic community, which he defines as a group of professionals who share:

- a set of normative and principled beliefs, which provide a value-based rationale for the social action of community members;
- causal beliefs, derived from their analysis of practices to address problems in their domain, that serve as the basis for understanding linkages between possible policy actions and desired outcomes;
- notions of validity, including criteria for weighing and validating knowledge in the domain of their expertise; and
- a set of common practices associated with the problems to which their professional competence is directed, with a view to enhance welfare.

A common feature of successful examples of international cooperation in different regulatory policy areas is the existence of an epistemic community. Haas is interested in epistemic communities precisely for the way in which they ease international cooperation in policy domains characterized by a substantial degree of technical knowledge that can form part of the basis for such cooperation. At this point in time, no such epistemic community exists around the international regulation of GVCs.

One mechanism for bringing together an epistemic community is what is sometimes called a knowledge platform, a forum aimed at fostering a substantive, evidence- and analysis-based discussion of the impacts of sector-specific regulatory policies (Hoekman, 2014), which involve the participation of businesses, officials (such as trade policy officials) and regulators.^16^ Analysts are present to add value to the policy information provided but also to assist in the management of the risk of loss of attention to the public interest. The objectives of such platforms are consistent with approaches that have long been pursued by some inter-governmental organizations. APEC and the OECD are examples of entities that already provide an institutional home for this type of engagement.

**Figure 3:**
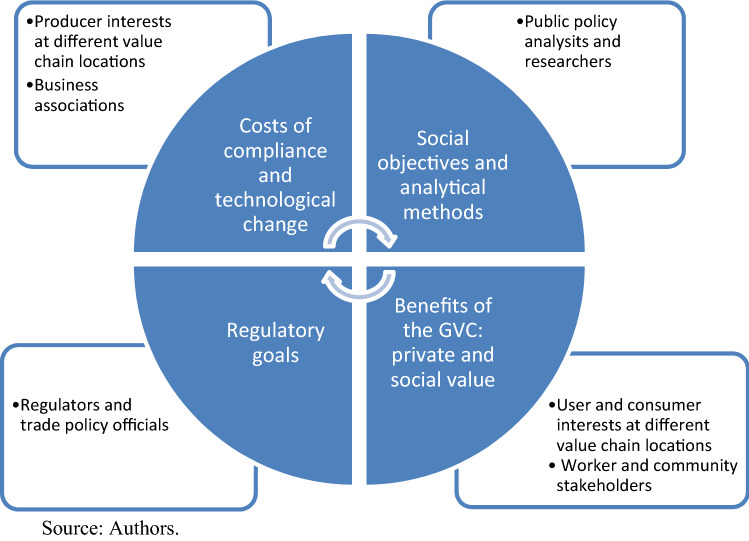
A value chain partnership.

The scope of interest and therefore participation in what we refer to hereafter as a value chain partnership is summarized in Figure 3.^17^ The figure is intended to reflect the forces at work in political economy processes linked to policy change, since as we have noted there is a range of interests to be represented in this activity. The suppliers of policy change are the regulators and officials at the bottom left. They explain the purpose of the policy under consideration, for example, the market failure that is being resolved. The demanders include the participants at the top left and bottom right, producers and consumers (or users), as well as other stakeholders such as workers and community representatives. The sources of value in the chain (private and social) are identified in a dialogue led by participants in the bottom right. Producers at the top left identify the impediments to capturing that value, also with some attention to technological change in use and in production process. In a value chain setting, these participants include producers and users at various points of value adding (some producers at one point in the chain are users with respect to earlier points). In a global value chain context, there will be a variety of participants across countries (the figure can be thought of being stacked vertically by country to complete the picture). At the top right are public policy analysts and researchers who maintain a focus on the social objectives, and who provide research and analysis capacity to facilitate the resolution of the discussion with respect to the direction of policy change across countries.

The construction of these arrangements is anchored locally, either at sub-national and/or national level, but these partnerships must be connected to counterparts offshore since that is where some of the constraints may lie. There are various options for establishing the international connections. In some cases, national entities will be aware of relevant counterparts. Another mechanism is via the networks associated with trade agreements, both bilateral and regional. Some agreements now contain cooperation chapters, which provide a framework and funding for this purpose.^18^ Regional-level bodies can also provide advice on participation: APEC for example has a committee on standards and conformance which could contribute as a clearing house of information.^19^ Where regional structures were not available, another option could be based on plurilateral structures developed in the WTO (Hoekman & Mavroidis, 2015; Hoekman & Sabel, 2019), where cooperation could be organized according to principles of transparency, openness, and inclusiveness. Commitments to and processes established under the WTO Trade Facilitation Agreement are relevant. Some more specific examples of how to proceed are offered in the next section.

## EXAMPLES OF OPERATIONALIZING THE CONCEPT

To the best of our knowledge, there are no extant value chain partnerships or platforms that encompass all the various elements discussed above. There are, however, many examples of initiatives that address some dimensions of what such a body might do and that bring together many of the relevant stakeholder groups affected by or concerned with the operation of value chains. Many countries have trade and investment promotion bodies aimed at increasing and diversifying exports (moving along the extensive margin and upgrading participation in GVCs). Many countries also have put in place mechanisms to facilitate imports – aimed at reducing the transactions costs associated with moving goods across borders and along transport/transit corridors. Such mechanisms are by design multi-sectoral and bring together public and private actors, but generally do not reflect the value chain nature of international production, nor do they encompass noneconomic issues in the way that multi-stakeholder value chain sustainability partnership initiatives do (Mohan, 2018; Soundararajan, Brown, & Wicks, 2019). As mentioned, the latter are often organized around a specific product or sector and have a focus on standard-setting and implementation. National trade promotion and facilitation bodies tend to take prevailing regulation in both the domestic and export markets as given.

There is an analogue here with modern approaches to industrial policy that rely on public–private dialogue mechanisms and institutions to identify and resolve coordination problems – viz Sabel (2012), Ross-Schneider (2015), and Devlin and Pietrobelli (2019). The aim is to foster communication between actors with a stake in an economic activity (value chain) and to work together to identify and address specific constraints (including market failures) that affect the feasibility and profitability of a new activity. Information on weak links and missing complementary inputs is located at the level of firms and local authorities and stakeholders.

Value chain partnerships can build on and learn from these types of initiatives, combining the attention on value chains and trade facilitation with a focus on analysis of how policies – or a lack of policies – may unnecessarily raise costs and efforts to define actions – including international cooperation – to reduce trade costs. Illustrative examples of this approach are presented in Bain and Co (2014) in case studies of several specific agricultural export value chains – cassava in West Africa; tomatoes (India) and avocados (Kenya) – that identify how public–private collaboration was important in identifying factors where policy reforms and cooperation between actors reduced inefficiencies and increased the benefits of GVCs to participants. The case studies demonstrate how dialogue informed by analysis can identify both policy-related frictions that detrimentally affect vertical dimensions (stages) of specific value chains and/or impact on activities that are important for many different value chains. Solutions often will involve several government departments within a country, possibly multiple levels of government, and, because of the GVC nature of production and exchange, cut across several countries.

Following the conclusion of negotiations in 2013 to establish the WTO Trade Facilitation Agreement (TFA), a major locus for public–private cooperation to reduce international trade costs has centered around trade facilitation initiatives. Such cooperation has tended to take two forms. One is policy dialogue. An example is the Africa Trade Facilitation Forum (ATFF), organized by the African Union Commission with support of international organizations and donor countries in 2016. The main objective of the ATFF is to provide a platform for policy dialogue between trade officials, executives from the private sector, development banks and donor agencies, parliamentarians, and civil society.^20^ Another form is more operational, and focuses on implementing measures to facilitate trade on the ground. An example is the Global Alliance for Trade Facilitation, a 2015 World Economic Forum initiative hosted by the International Chamber of Commerce. A collaboration between businesses, governments, and international organizations, the alliance seeks to assist governments in developing countries implement the TFA through partnerships between governments and businesses to address delays and unnecessary red tape at borders through jointly designed reforms and projects that deliver commercially quantifiable results. The alliance is implementing projects in Africa, Latin America, and Asia that address horizontal GVC trade facilitation frictions.^21^

The Pacific Alliance (Chile, Colombia, Mexico, and Peru) provides an example of cooperation between governments that includes both forms of public–private policy dialogue and concerted action. National leaders agree on areas for joint action which are then elaborated by the ministers of foreign affairs and foreign trade of each member state. A business council has a formal role in contributing suggestions on how to address frictions and differences in regulations that increase trade costs, in 2014 putting forward proposals on ways to harmonize technical standards in specific sectors such as cosmetics, pharmaceuticals, and processed food, and reducing time and cost of trade by making national single-windows systems interoperable (Inter-American Development Bank and Bain & Co, 2014). A High-Level Group is charged with monitoring progress made by technical working groups charged with implementing the action agenda defined by Ministers. The most effective forms of multi-stakeholder arrangements will involve a high-level monitoring and certification process: measurement and then accountability is critical for success.

A potentially significant building block in operationalizing the concept of value chain partnership is to build on a requirement of the WTO TFA to establish a national committee on trade facilitation (NCTF) to oversee and guide implementation of the agreement. Some 130 countries now have a NCTF.^22^ These bring together different government agencies that implement regulations applied at the border and include private sector representation, usually business associations or chambers of commerce. The TFA takes national regulations as given and calls on WTO members to put in place good practices in the process of determining compliance with regulatory requirements. However, nothing precludes governments from asking business representatives in NCTFs from using GVC-informed approaches to identify sources of frictions and suggest ideas for policy reform or international regulatory cooperation.^23^ Widdowson, Short, Blegen and Kashubsky (2018), noting the prevalence of these Committees, also observe that when joined together they (or similar national bodies) could focus on GVC operations (p. 33). Organizing a series of such interactions on various GVCs is a feasible first step towards the concept proposed here.

NCTFs or analogues already existed in many countries, but the fact that an international agreement (the TFA) requires such bodies is important in ensuring that they are functional as countries must regularly report on progress in implementing the agreement to the WTO.^24^ This “commitment device” role of trade agreements is valuable in helping to overcome standard political economy constraints to sustaining a focus on measures to enhance the operation of value chains. One such constraint is funding. The legal commitment to implement the TFA increases the likelihood that public resources will be allocated to the bodies to fulfill their function. Moreover, the TFA calls on donor countries and international organizations to support developing countries on request. As a result, development agencies created various support mechanisms to provide technical assistance and the relevant international agencies are coordinating their activities in this area – and are tasked with annually reporting on their work to the WTO Committee on Trade Facilitation.

The value of anchoring policy dialogue mandates in a trade agreement and including linkages to development cooperation is illustrated by a project described by Gray, Laukkala and Findlay (2020), which captures many of the features of a value chain platform. Its purpose was to discuss the standards, laws, and regulations affecting various products exported from Indonesia to Australia. The project was funded by the Australian government in association with the development of a bilateral trade agreement with Indonesia, which entered into force in July of 2020.^25^ The design of the project was consistent with relevant chapters in the trade agreement on technical barriers to trade and on cooperation. Participants included staff of regulatory bodies from both countries, businesses from the sectors covered (Food, Drugs and Herbal Well-being Products) and researchers, who also convened the activities. The work was organized around taking a value chain approach and linking to cooperation on regulatory reform, with participants reporting on specific bilateral transactions, and removing the impediments they faced.

Project outcomes included better understanding among exporters of consumer expectations of quality, safety and risk, and therefore how those expectations fed into the design of regulation, an appreciation of the mechanisms of regulation in the importing country, an understanding of options for reducing costs of compliance, a recognition of the consistency of the standards involved with those of international bodies and therefore how this cooperation would also facilitate trade with third countries. Gray et al. (2020) refer to reductions in compliance costs, lower failure rates in compliance, including with respect to labeling regulations, and success in establishing new distribution systems. There were examples of export growth following the project. Activities of the partnership structure proposed here were evident, for example, policy transparency, analysis of policy impacts, consultations on policy objectives, examination of alternatives and discussion of the origins of regulation, as well as sharing of experience and of good practice in the operation of regulation.

Lessons reported by Gray et al. (2020) include the value of linking the operation of a platform of this type to a trade agreement that provided guidance for the activity according to its principles. This created an incentive for completion of the work, as well as the resources required. They also observe the value in future occasions of considering more complex chains with more stages, and of including more interests associated with the downstream end of the chain, including distributors and consumers. Finally, they note the value of establishing a mechanism for regular joint reviews of policy that include input from the business community and civil society since circumstances (including in this case, the results of medical research or studies of nutrition) continue to change.

In summary, the model proposed can and should operate at various levels. It would start at the national level, for example, linked to the NCTF. These national platforms are then connected to international counterparts. This can be done under the umbrella of a regional organization, including a trade agreement, and better so with multi-country agreements, such as those in ASEAN or the CPTPP. Other options vary between regions. In the Asia-Pacific, APEC has a role. In the absence of other regional structures, an option is to use an open plurilateral agreement, which could also be connected to the WTO. More than one structure could also be engaged, depending on their complementarities (e.g., implementation mechanisms may be linked with trade agreements but designed according to principles endorsed in APEC, with capacity-building also managed under the APEC umbrella).

An example that goes in this direction is the APEC Wine Regulatory Forum.^26^ This was set up in 2011 and operates under the APEC Sub-Committee on Standards and Conformance. One of its achievements was an APEC Model Wine Export Certificate reflecting a consolidation of existing requirements found in export certificates most frequently used in wine trade among the APEC economies, including certificates of origin, authenticity/free sale, and health/sanitation. The idea emanated from a consolidated wine document that the People’s Republic of China (China) and the United States established in 2014.^27^ Interviews with participants in this forum indicate that it has been effective in inducing the withdrawal of redundant requirements. An example was a limit on manganese in wine imposed by China that was withdrawn after a presentation on the safety of prevalence of naturally occurring substances; another was a decision by the Philippines to regulate wine as a “low-risk” food, reducing testing requirements. The forum promoted the adoption of E-certificates for minimum residue levels. A similar mechanism is now being established by the dairy industry.^28^ According to the framework developed here, directions for the development of the forum include taking an explicit value chain view, confirming and extending private sector participation from that perspective, adopting a reporting system on targets and outcomes, and considering forms of engagement with public policy participants.

To be effective, value chain platforms require active engagement by international business, working through associations that are representative of a broad cross section of sectors. Given the value chain focus, logistics industry associations may be well placed to take a leading role, supported financially and technically by firms and industry groups that rely heavily on international sourcing of inputs and export a significant share of their production. A question that must be resolved is the scope of the value chain to be considered, and therefore the range of participants. Input from the stakeholders is important, especially businesspeople, since they will be aware of the associated sets of linkages between producers and the relevant degrees of substitutability, either in demand or supply. Government has a role in convening the platform, but the focus of the platform would be a result of guidance from stakeholders. That scope may also change over time as technology shifts. In this paper, an example of the face mask GVC was provided. This may be the focus of a platform of the type proposed here, but also the advice from stakeholders may be that a wider view of all personal protective equipment is valuable.

Another dimension of scope that demands attention is the approach to the assessment of regulatory measures. Success is more likely if the methodology of the platform is to work from the top and down, rather than working from the bottom and up, that is, measure by measure.^29^ The starting place of the work of the platform could be to compare the purposes of the regulation in different jurisdictions, followed by a characterization and comparison of the processes that are used to specify regulations for those purposes. Then might follow some consideration of instances of regulation or measures, but otherwise an alignment of principles and processes would be expected to guide change at the economy level. The documentation and analysis of this methodology and the sharing of the experience is another function of the research community participants.

A commitment to setting goals for progress and monitoring performance will be critical for success. Various indicators of value chain performance could be used, some of which are physical measures, such as those related to inventory management^30^ or the speed of movement or ‘velocity’ of goods in the chain. Others are indices of risk (Mysore & Usher, 2020). Also of interest, however, are the costs of managing the process of value adding, which depends on the responses of chain participants to the performance indicators. Further research is important, including that which develops methods of mapping trade costs within value chains to both regulatory policies and to these physical measures. Research could be undertaken to measure these relationships. Doing so would also help build the case for cooperation. Business can play a role by sharing data and experience. The amount of data available is likely to rise with the digitization of the processes associated with value chains (Schniederjans, Curado, & Khalajhedayati, 2020). International business scholars can also play a role by transforming the information provided by firms into indicators that can be used for monitoring and assessment. Platform performance, and innovation, then depends on reporting back to participants and platform managers on these outcomes.

These sorts of activities will entail costs, but these are relatively limited – essentially time associated with consulting on policy-induced sources of value chain frictions with participating firms, compilation of performance measures (e.g., indicators) and participating in the deliberations of the partnerships. The return on investment is likely to greatly exceed the costs, and the extent to which this is the case can (and should) be assessed periodically. Costs for government officials are essentially limited to participation (time and travel). As noted, anchoring or linking the activity to a trade agreement will facilitate allocation of the requisite budgetary resources. Civil society groups will need to fund their participation and for them the same premise applies as for business: the proof of the pudding is in the eating. The plethora of multi-stakeholder sustainability initiatives suggests that financing is unlikely to be a binding constraint. One cost that will need to be shared by the core of the public–private partnership – business and government – is the research and analysis that is needed to inform effective deliberation. Here again, overall resource requirements will not be great, especially if business is willing to work with analysts to identify and compile data on value chain frictions.

For many developing countries, official development assistance is a potential source of funding to cover some of the costs of operating value chain platforms at the national level as well as participation in regional or international meetings. Recent experience has demonstrated the feasibility of international meetings using video conferencing, greatly reducing the cost of international engagement between national platforms. The global aid for trade initiative is a source of funding to support activities in developing countries, given the goal of such aid is to promote trade and upgrading the capacity to engage value chain-based activities.^31^ Using aid for trade funds to support value chain platforms can both increase the ‘rate of return’ and improve coherence between trade policy and implementation of trade agreements and development policies.

## LEARNING FROM COVID-19 POLICY RESPONSES

**Figure 4:**
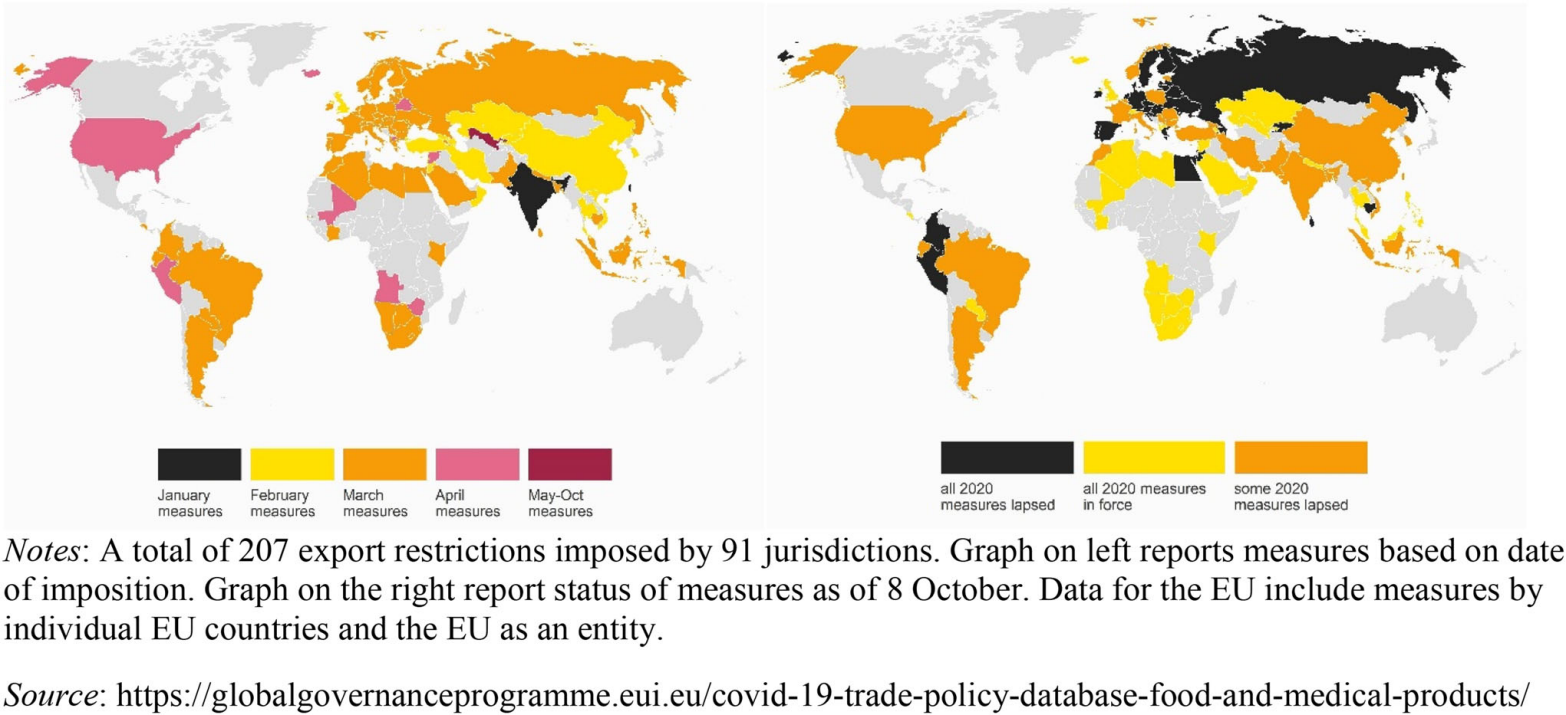
Export controls and medical supplies from January to October 8, 2020.

The COVID-19 pandemic illustrates the potential value of public–private value chain-centered cooperation. The pandemic led to a rapid increase in demand for personal protective equipment and medical supplies. Despite the entry of many new suppliers and the ramping up of output by established producers,^32^ many countries resorted to export restrictions to maximize domestic supply (Figure 4). A project by the EUI, the Global Trade Alert, and the World Bank to monitor the use of trade policy responses to the pandemic reveals that the intensity and duration of such restrictions varied extensively across countries. Some EU countries, such as France, banned exports early in the pandemic. Some of the large emerging economies (e.g., Brazil and India) imposed many measures. In many cases, the most restrictive measures were temporary and were withdrawn in the course of several months, but as can be seen from the right-hand graph in Figure 4, several countries still maintained export controls 8 months after the start of the pandemic.

Export controls are counterproductive, as they can exacerbate shortages of key inputs supplied by foreign firms (see Figure 2), especially if they lead to tit-for-tat retaliation or emulation. Reshoring production is not the lesson to draw from the COVID-19 pandemic, as that would be a costly exercise that is unlikely to improve availability of supplies when the next global demand and/or supply shock hits. What is needed is to enhance the ability to source supplies rapidly and for firms, no matter where they are located, to respond.^33^

To ramp up production of essential protective and medical products, companies need information on demand, applicable health, and safety standards, be able to obtain rapid certification, and to source requisite inputs – including from foreign suppliers. Effective two-way communication channels are needed for firms to identify specific bottlenecks that impede the ramping up of supply. Companies need to have systems to monitor market conditions and identify slack and chokepoints in their global network to enable adjustments in production to respond to changes in demand (Alicke, Azcue, & Barriball, 2020). Governments need information systems that allow them to determine where supply capacity exists and how goods are produced. Firms generally will have information on supply options, but governments often will not have such information readily on hand. Both sets of actors need to be able to identify bottlenecks in real time and cooperate in addressing them. The platforms proposed here provide a framework for that cooperation. The basic data concerning the performance of the chain, and the value of adjustments to regulatory processes, is relevant for this purpose. Those same indicators, and the same (increasingly digital) data collection systems, can be applied to assessments of resilience as well (Miroudot, 2020; Singh, Soni, & Badhotiya, 2019). A value chain platform for personal protective equipment and medical supplies, including vaccines, for example, would help identify sources of friction impeding production expansion that are due to – or can be overcome – through policy action as well as measures to improve resilience.

Such information systems were not in place in many if not most countries when COVID-19 struck in 2020. Many authorities did not have a good understanding of the prevailing value chains and their production capacity. There is a notable contrast here with agricultural products, where traceability has become a common feature of the production and distribution process, and where multi-stakeholder partnership initiatives have helped stakeholders – including governments – better understand the organization and operation of the associated value chains.

As discussed in “Regulatory Heterogeneity, International Trade Costs, and GVCs”, standards and certification of products/plants/suppliers are critical for safety, but the associated regulatory enforcement processes can not only be an unnecessary source of trade costs but also a constraint in the ability of companies around the world to respond rapidly to an emergency. The existence of common product standards and mutual recognition of standards facilitates supply responses and the operation of cross-border production arrangements. The opportunity costs of not having equivalence and recognition regimes in place were illustrated by the decision by China to impose new export license requirements in early April 2020 (Hoekman, Fiorini, & Yildirim, 2020). The government was responding to rejections by several European countries of PPE shipments sourced from Chinese companies on quality grounds. The Chinese authorities feared a reputational backlash and sought to ensure that exported products meet quality and safety standards by limiting exports to firms certified to sell in domestic markets (i.e., firms having been accredited as meeting Chinese technical regulations). Companies that only produced for export and were accredited in the US or EU – e.g., firms with FDA and CE certification – were constrained from exporting by the new regulation until they had obtained certification in China.^34^

Cooperation between governments (regulators) to establish recognition and equivalence arrangements for certification and acceptance of foreign standards would help not only in reducing trade costs (“Regulatory Heterogeneity, International Trade Costs, and GVCs”) but have the added benefit of supporting access to supplies in a time of crisis. Value chain platforms that facilitate information exchange and regulatory cooperation would have helped both governments and industry understand the state of play and coordinate policy responses, address bottlenecks, and strengthen supply responses.

## CONCLUSION

The operation of GVCs offers important opportunities for business, workers, and consumers in both developed and developing countries. These are evident in the experience to date, including facilitating the entry into complex manufacturing or services production systems, encouraging inward flows of capital and technology, expanding employment in higher skilled jobs, leading to rising productivity and to income growth. These benefits arise from relatively complex processes, in which policies in the participating countries can affect the costs of doing business and the design of the chains. Trade costs can sometimes be avoided by reconfiguring the chains, although not without incurring other costs. There is benefit, therefore, in businesses cooperating with other stakeholders to identify and tackle policy impediments to the efficient operation of GVCs. Given the diversity of the policies involved, this effort demands new forms of cooperation to address the spillover effects of national policies on the operation of GVCs.

As noted in the introduction to this paper, a variety of factors are driving changes in global value chains. These forces include the application of policy. There is an increasing prospect for the consolidation of value chains, reflecting industrial policy and/or economic/national security considerations, including concerns about the risk of disruptions to the operation of international production networks through natural disasters and pandemics. Recent trends and developments bolster the case for public–private cooperation around GVCs. The COVID-19 pandemic greatly increased public awareness and political scrutiny of GVCs for medical supplies and protective equipment, and concerns regarding the resilience and robustness of the associated production networks. Mechanisms to provide greater insight and information on the design and operation of GVCs and to facilitate coordination between business and governments could have helped support the necessary ramping up of global production instead of disrupting key value chains by use of export controls. Public–private cooperation around GVCs would help counteract political pressures for reshoring and ‘strategic autonomy’ characterized by a return to vertically integrated national production of “essential supplies” – something that is difficult to circumscribe. More generally, such cooperation would help ensure a focus on efficiency (cost reduction) without undercutting the realization of the objectives that underlie national regulation.

Developing economies have perhaps the greatest incentive to work with international business to take a lead role in this work. The situation was once much in the favor of developing economies, as their trading partners sought to engage them in global value chains. Yet global value chains have become important for developing countries. The fight against the opposite flow could be built on an effort to tackle the policy impediments which raise the costs of their participation, thereby helping to offset the incentives to reshore created by technological and other changes.

## Notes

1. E.g., the FAO Sustainable Food Value Chains Knowledge Platform (http://www.fao.org/sustainable-food-value-chains/home/en/); the OECD Initiative for Policy Dialogue on Global Value Chains, Production Transformation and Development (https://www.oecd.org/dev/GVC_Initiative_Brochure_2015-01.pdf); the World Bank’s Global Partnership for Social Accountability Knowledge Platform (https://gpsaknowledge.org/); and the UN Global Compact on Supply Chain Sustainability (https://www.unglobalcompact.org/engage-locally/manage/engagement/supply-chain-sustainability).
2. Bakker, Rasche and Ponte (2019) review the literature on multi-stakeholder initiatives from a multi-disciplinary perspective.
3. Here we focus on the operation of value chains. As distinct from a supply chain, the concept of a value chain was proposed in the development studies literature by Gary Gereffi and co-authors – see e.g., Gereffi, Humphrey and Sturgeon (2005) – to emphasize the importance of considering issues such as design and branding that add value to a product and to recognize that value is created by workers and suppliers of inputs and ancillary services. The international economics and economic geography literatures often use the term global production networks instead of value chains in recognition of the fact that production often encompasses a network of firms in different countries. We mostly use the term global value chain because it has become prevalent in policy discussion, but it largely overlaps what is understood by global production networks. See, e.g., Coe (2014).
4. As noted below, there is a close parallel to modern approaches to industrial policy and innovation, which centers around addressing coordination problems through public–private dialogue and joint problem solving (Sabel, 2006).
5. Fajgelbaum, Goldberg, Kennedy and Khandelwal (2020) identify the significance of this trend in the US economy.
6. Hernandez and Pedersen (2017) review research on the concept of the global value chain, forms of its configuration and its management from an international business perspective; Kano, Tsang and Yeung (2020) offer a comprehensive review of the literature across disciplines.
7. The 2020 World Development Report (World Bank, 2020) summarizes much of the literature and evidence on the effects and drivers of GVCs.
8. UNCTAD (2013) focuses on links between GVCs and FDI (multinational corporations).
9. This example refers to both N95 respirators and surgical masks. Their features are summarized by the US Centers for Disease Control and Protection at this link https://www.cdc.gov/niosh/npptl/pdfs/UnderstandingDifference3-508.pdf.
10. Gereffi (2020) observes that the point at which there is greatest risk of a bottleneck in this chain is at the production of the melt-blown polypropylene which is produced by a small number of companies worldwide and which involves specific and capital-intensive processes, impeding the ability of other producers to switch quickly into the production of this input. See also OECD (2020).
11. Where compliance cost is denoted by C and the number of stages by n then the relationship of total compliance cost to stages is C = 0.5n^2^ + 0.5n + 4. The rate at which cost is changing at a given stage is n + 0.5, which increases with the number of stages. Even though the cost of compliance is considered here as a fixed cost, the burden of compliance increases with the number of stages. Other research has stressed the exponential effect of trade costs, which are a proportion of the value of the item moving through GVCs (Ferrantino, 2012).
12. The discussion here assumes events in the GVC occur in a sequence. Were this chain organized in a network, where design and components are brought together from two origins direct to the assembly stage in a third economy followed by distribution in the (fourth) destination country the compliance cost is reduced, since the design has only to be confirmed in two countries rather than three.
13. https://www.globalgap.org/uk_en/.
14. In many cases, deliberations of these bodies are informed by interactions with international business associations – the B-20 in case of the G-20; the APEC Business Advisory Council and Industry Dialogues in the APEC case and the Business and Industry Advisory Committee in the OECD. Other second track bodies can also play a role, as in the relationship of the tri-partite Pacific Economic Cooperation Council with APEC.
15. Over 50% of business respondents to the Pacific Economic Cooperation Council’s State of the Region surveys for 2019–20 and 2018–19 regarded the WTO and the multilateral trading system a top-5 topic for attention by leaders of the members of the APEC economies, compared to 11% in the 2071–18 report (PECC, 2019, Figure 1.29).
16. See footnote 1 for examples.
17. We use the term platform or partnership interchangeably.
18. An example is chapter 12 of the ASEAN Australian New Zealand Free Trade Agreement. See https://aanzfta.asean.org/chapter-12-economic-co-operation/. A work program is agreed by the FTA Joint Committee and focal points are identified and responsible for its implementation. Resources are provided by the parties.
19. See https://www.apec.org/Groups/Committee-on-Trade-and-Investment/Sub-Committee-on-Standards-and-Conformance.
20. https://www.uneca.org/sites/default/files/uploaded-documents/ATPC/ATW2016/atff-programme_november-26th.pdf.
21. Private sector participation includes Fiat Chrysler Automobiles, UPS, Maersk, insurer Roanoke Trade (part of Munich Re), BASF and frozen foods vendor ArdoVLM. See https://tradefacilitation.og.
22. https://unctad.org/en/DTL/TLB/Pages/TF/Committees/default.aspx.
23. See Hoekman (2014) and Belastegui (2020). See Widdowson et al. (2018) on NCTFs more generally.
24. Similarly, trade agreements are an important focal point for export and investment promotion bodies, providing a framework for engaging with authorities on trade matters.
25. https://www.dfat.gov.au/trade/agreements/in-force/iacepa/Pages/indonesia-australia-comprehensive-economic-partnership-agreement.
26. See https://www.apec.org/Achievements/Group/Committee-on-Trade-and-Investment-2/Sub-Committee-on-Standards-and-Conformance.
27. https://www.wineregulatoryforum.org/honolulu.
28. Interviews held in September 2020.
29. Officials in APEC working on food standards have moved ‘beyond the use of detailed side-by-side comparisons of specific inspection and certification measures in favor of a transparent, top-down approach that looks at the design of the system as a whole or relevant parts of it. “Attempts by regulators to determine equivalence between food safety systems can quickly become overwhelmed by the expansiveness of a bottom-up, measure-by-measure approach,” said Dr. Bill Jolly, Chief Assurance Strategy Officer of New Zealand’s Primary Industries Ministry. “System equivalence assessments and associated equivalence agreements that enhance cooperation between competent exporting and importing market authorities are likely to be more efficient and effective,” “Ultimately it’s about putting risk in perspective.” Extracts from: https://www.apec-vc.or.kr/Default.asp?p_name=newsroom&sort=NA&gotopage=2&query=view&unique_num=9332).
30. See for example https://www.uspsdelivers.com/10-kpis-that-can-help-improve-your-inventory-management-process/.
31. World Bank (2020). For an overview of the activities under the Aid for Trade initiative and biannual reports and information on aid for trade programs, see https://www.wto.org/english/tratop_e/devel_e/a4t_e/aid4trade_e.htm. This includes a focus on GVCs – see e.g., OECD and WTO (2013).
32. https://www.detmoldgroup.com/detmold-medical/.
33. Adjustments may include re-shoring, but also value chain structures may be diversified: see DuHadway, Carnovale and Hazen (2019) for a review of types of risks and responses.
34. To avoid such a situation, the Korean Ministry of Trade, Industry and Energy inspected 113 makers of melt-blown fabric in 33 countries before contracting with two foreign suppliers for this key component of face masks. Mutual recognition would reduce such transactions costs. https://world.kbs.co.kr/service/news_view.htm?lang=e&Seq_Code=152173.
